# Reclassification of *Parapterulicium* Corner (Pterulaceae, Agaricales), contributions to Lachnocladiaceae and Peniophoraceae (Russulales) and introduction of *Baltazaria* gen. nov.

**DOI:** 10.3897/mycokeys.37.26303

**Published:** 2018-07-31

**Authors:** Caio A. Leal-Dutra, Maria Alice Neves, Gareth W. Griffith, Mateus A. Reck, Lina A. Clasen, Bryn T. M. Dentinger

**Affiliations:** 1 Micolab, Departamento de Botânica, Centro de Ciências Biológicas, Universidade Federal de Santa Catarina, Florianópolis, Santa Catarina, 88040-900, Brazil Aberystwyth University Aberystwyth United Kingdom; 2 Institute of Biological, Environmental and Rural Sciences, Aberystwyth University, Aberystwyth, Ceredigion SY23 3DD, UK Universidade Federal de Santa Catarina Santa Catarina Brazil; 3 Natural History Museum of Utah and School of Biological Sciences, University of Utah, Salt Lake City, UT, 84108 USA CAPES Foundation, Ministry of Education of Brazil Brasília Brazil; 4 CAPES Foundation, Ministry of Education of Brazil, P.O. Box 250, Brasília – DF 70040-020, Brazil University of Utah Salt Lake City United States of America

**Keywords:** Molecular Phylogeny, Taxonomy, Russulales, /peniophorales, Corticioid fungi, Coralloid fungi

## Abstract

The genus *Parapterulicium* was first introduced to accommodate two Brazilian species of coralloid fungi with affinities to Pterulaceae (Agaricales). Despite the coralloid habit and the presence of skeletal hyphae, other features, notably the presence of gloeocystidia, dichophyses and papillate hyphal ends, differentiate this genus from Pterulaceae*sensu stricto.* Fieldwork in Brazil resulted in the rediscovery of two coralloid fungi identifiable as *Parapterulicium*, the first verified collections of this genus since Corner’s original work in the 1950s. Molecular phylogenetic analyses of nrITS and nrLSU sequences from these modern specimens revealed affinities with the /peniophorales clade in the Russulales, rather than Pterulaceae. The presence of distinctive hyphal elements, homologous to the defining features of /peniophorales, is consistent with the phylogenetic evidence and thus clearly distinguished *Parapterulicium* and its type species *P.subarbusculum* from Pterulaceae, placing this genus within /peniophorales. *Parapterulicium* was also found to be polyphyletic so *Baltazaria* gen. nov. is proposed to accommodate *P.octopodites*, *Scytinostromagalactinum*, *S.neogalactinum* and *S.eurasiaticogalactinum* also within /peniophorales.

## Introduction

Pterulaceae Corner is a diverse but poorly known family of mostly tropical coralloid fungi within order Agaricales Underw. (Dentinger and McLaughlin 2006), recognised mainly by their coralloid/filiform basidiomes with a dimitic hyphal structure (Corner 1952a, 1952b, 1957, 1970).

To date, only three of the five Pterulaceae genera have been included in molecular phylogenetic analyses, viz. *Pterula* Fr., *Deflexula* Corner and *Pterulicium* Corner (Dentinger and McLaughlin 2006, Dentinger et al. 2009). The remaining genera, *Parapterulicium* Corner and *Allantula* Corner, are known only from a few scant specimens collected by Corner as the basis of his taxonomic proposal; these are poorly preserved and impractical for molecular studies. *Allantula* (meaning ‘sausage’ in ancient Greek), characterised by decumbent, intercalary swellings resembling minute sausages, is known only from the type specimen (Corner 1952a) and has not been recollected despite several recent attempts at the type locality (Parque Nacional da Tijuca, Rio de Janeiro, Brazil) by the present authors. *Parapterulicium* was described for two coralloid species from Brazil that resembled Pterulaceae in their filiform statues and dimitic hyphae, but differed in the presence of gloeocystidia and dichophyses.

Corner (1952a) suggested some similarity of *Parapterulicium* to *Lachnocladium* Lév. based on the shared features of dichophyses and gloeocystidia combined with the lack of clamps. However, due to the small filiform basidiomes, branching pattern, colourless dimitic hyphae and corticioid patch, Corner referred the genus to Pterulaceae instead of Lachnocladiaceae. Corner’s emphasis of skeletal hyphae as a synapomorphy for Pterulaceae has been shown previously to be incorrect with the reclassification of *Actinceps* Berk. & Broome (=*Dimorphocystis*) (Dentinger and McLaughlin 2006), although this feature remains a defining feature of Pterulaceae.

During recent field expeditions in four Brazilian states, two coralloid fungi morphologically assignable to *Parapterulicium* spp. were collected, providing fresh material for molecular phylogenetic analysis. Here we present results that show *Parapterulicium* is paraphyletic and evolutionarily related to Peniophoraceae Lotsy and Lachnocladiaceae D.A. Reid in the Russulales Kreisel ex P.M. Kirk, P.F. Cannon & J.C. David, rather than Pterulaceae in the Agaricales. We propose taxonomic changes precipitated by these results and provide a re-evaluation of distinctive morphological features, such as variations in skeletal hyphae that may be considered phylogenetically informative in light of this discovery.

## Methods

### Collections and morphological observations

The new collections of *Parapterulicium* are deposited at FLOR, INPA and RB. Herbarium acronyms follow Index Herbariorum (Thiers continuously updated). Macroscopic analyses were conducted following the traditional methods of Largent (1986).

Microscopic analyses were adapted from Largent et al. (1977) for pterulaceous fungi, where, instead of sectioning the basidiomes with a razor, part of the fungus was dissected with the aid of two small diameter needles. The dissections were mounted in 5% KOH, H_2_O, Melzer’s reagent, Congo red or 1% phloxine and then observed with an Olympus CX21 (Olympus, Tokyo, Japan) light microscope equipped with 10x, 40x and 100x objective lenses, the latter being used with immersion oil. Macro- and microscopic illustrations were based on pictures taken in the field with a Nikon D90 DSLR camera (Nikon, Tokyo, Japan) and on photos taken during microscopic observations. The colour codes follow the Munsell Soil Color Charts (Munsell 1975). Scanning electron microscopy (SEM) images were obtained using a Hitachi S-4700 field emission scanning electron microscope (Hitachi, Tokyo, Japan) and the air-dried specimens were directly stuck on the carbon tab on the stubs without any treatment. The stubs were coated with gold and platinum and examined and photographed at 5 kV.

### DNA extraction, PCR amplification, cloning and sequencing

DNA was extracted from dried basidiomes by first grinding with a mortar and pestle in the presence of liquid nitrogen, followed by purification using the DNeasy Plant Mini Kit (Qiagen) according to the manufacturer’s instructions. Partial sequences of the nuclear ribosomal internal transcribed spacers (nrITS) and nuclear ribosomal large subunit (nrLSU) were amplified by PCR using the primer pairs ITS8F-ITS6R (Dentinger et al. 2010) and LR0R-LR7 (Vilgalys and Hester 1990), respectively and following the cycling conditions in the original publications. PCR products were purified using 2 units of Exonuclease I (Thermo Fisher Scientifics) and 1 U FastAP Thermosensitive Alkaline Phosphatase (Thermo Fisher Scientifics) per 1 µl of PCR product, incubated at 37 °C for 15 min, followed by denaturing at 85 °C for 15 min. The samples were then sent for Sanger sequencing at the IBERS Aberystwyth Translational Genomics Facility.

Sequences and chromatograms were checked, assembled and edited using GENEIOUS 10.0.2 (Kearse et al. 2012). Samples presenting indels were cloned using pGEM-T Easy Vector Systems (Promega) into Subcloning Efficiency DH5α Competent Cells (Invitrogen). Five clones from each PCR were then amplified and sequenced as above. The sequences generated in this study have been submitted to GenBank (Table 1).

### Phylogenetic analysis

Prior to the inclusion in the datasets, the clones were aligned to generate one or two consensus sequences of each cloned species. Substitutions were replaced by the respective ambiguous code and, in the cases where indels were found, two different sequences were generated.

To assess the global phylogenetic position of *Parapterulicium* within Agaricomycetidae, a dataset containing the nrLSU sequences of 886 Agaricomycetidae taxa was created by adding the sequences generated in this study to the dataset of Moncalvo et al. (2002), as adapted by Dentinger and McLaughlin (2006). The analyses of this dataset demonstrated the placement of *Parapterulicium* within the Russulales. See Suppl. material 1: Agaricomycetidae analysis, for details and results of these analyses.

A more focused dataset for higher resolution phylogenetic analysis was created by removing duplicate species from the Russulales dataset of Chen et al. (2016) and adding the new sequences generated in this study alongside 29 GenBank sequences and one from CBS-KNAW database to represent all currently recognised families of Russulales, as well as all the genera of Lachnocladiaceae and Peniophoraceae with sequences available. Four sequences of *Sistostrema* Schumach. were used as outgroup. The Russulales dataset contained 135 sequences and was divided in four partitions: ITS1, 5.8S, ITS2 and nrLSU. A list of accession numbers of the sequences added to Chen et al. (2016) dataset is presented in Table 1; the complete list can be found in Suppl. material 1: SuppTable 1 and in Dentinger and McLaughlin (2006) for the Agaricomycetidae dataset.

The ITS1 and ITS2 datasets were aligned using MAFFT v7.311 (Katoh and Standley 2013) using the E-INS-i algorithm and the 5.8S and nrLSU datasets were aligned using the L-INS-i algorithm in MAFFT. The alignments were examined and adjusted manually using MEGA 7 (Kumar et al. 2016) and trimmed to remove uneven ends.

The best-fit evolutionary models were estimated for each partition separately using JMODELTEST v2.1.3 (Darriba et al. 2012; Guindon and Gascuel 2003) following the Bayesian Information Criterion (BIC). Bayesian Inference (BI) under the best-fit models was implemented using MRBAYES v3.2 (Ronquist et al. 2012) with two independent runs, each one with four chains and starting from random trees. Chains were run for 10^7^ generations with tree sampling every 1000 generations. The burn-in was set to 25% and the remaining trees were used to calculate a 50% majority consensus tree and Bayesian Posterior Probability (BPP). The convergence of the runs was assessed on TRACER v1.7 (Rambaut et al. 2018) to ensure the potential scale reduction factors (PSRF) neared 1.0 and the effective sample size values (ESS) were sufficiently large.

Maximum-likelihood analysis was performed with IQTREE v1.6.3.b (Nguyen et al. 2015). The best-fit evolutionary models for this analysis were estimated by the built-in ModelFinder (option -m MF+MERGE) allowing the partitions to share the same set of branch lengths but with their own evolution rate (-spp option) (Chernomor et al. 2016; Kalyaanamoorthy et al. 2017). Branch support was assessed with 1000 replicates of ultrafast bootstrapping (Hoang et al. 2018).

Nodes with BPP ≥0.95 and/or BS ≥75 were considered strongly supported.

Alignments and phylogenetic trees are deposited in Treebase (ID: 22642).

**Table 1.** Species from clade /peniophorales and their GenBank accession numbers of ITS and nrLSU sequences. Newly generated sequences are shown in bold.

Taxa	Sample no.	Locality	GenBank Accession no.		Reference
			nrITS	nrLSU	
* Asterostroma cervicolor *	KHL9239	Puerto Rico	AF506408	AF506408	Larsson and Larsson (2003)
* Asterostroma macrosporum *	TMI 25697	Japan	NR119394	–	Suhara et al. (2010)
* Asterostroma muscicola *	TMI 25860	Japan	AB439551	AB439551	Suhara et al. (2010)
* Baltazaria eurasiaticogalactina *	CBS 666.84	France	–	AY293211	Binder et al. (2005)
* Baltazaria galactina *	NH4863	Sweden	AF506466	AF506466	Larsson and Larsson (2003)
* Baltazaria neogalactina *	CBS 758.86	France	–	–	Unpublished
* Baltazaria octopodites *	FLOR 56442	São Paulo – Brazil	**MH260024**	**MH260043**	This study
				**MH260044**	
				**MH260045**	
				**MH260046**	
* Baltazaria octopodites *	FLOR 56449	São Paulo – Brazil	**MH260025**	**MH260047**	This study
* Baltazaria octopodites *	FLOR 56460	Santa Catarina – Brazil	**MH260032**	**MH260050**	This study
* Baltazaria octopodites *	FLOR 63715	Paraná – Brazil	**MH260042**	**MH260060**	This study
* Baltazaria octopodites *	INPA 280140	Amazonas – Brazil	**MH260038**	**MH260056**	This study
			**MH260039**	**MH260057**	
			**MH260040**	**MH260058**	
			**MH260041**	**MH260059**	
* Confertobasidium olivaceoalbum *	FP90196	USA	AF511648	AF511648	Larsson and Larsson (2003)
* Dendrophora albobadia *	TDeAB1029	USA	AF119522	AF119522	Hsiau and Harrington (2003)
* Dichostereum durum *	FG1985	France	AF506429	AF506429	Larsson and Larsson (2003)
* Dichostereum effuscatum *	GG930915	France	AF506390	AF506390	Larsson and Larsson (2003)
* Dichostereum granulosum *	NH7137/696	Canada	AF506391	AF506391	Larsson and Larsson (2003)
* Dichostereum pallescens *	NH7046/673	Canada	AF506392	AF506392	Larsson and Larsson (2003)
* Duportella lassa *	SP6129	Russia	KJ509191	KJ509191	Spirin and Kout (2015)
*Entomocorticium* sp.	FL_19	USA	KJ620518	KJ620518	Bracewell and Six (2014)
* Gloeocystidiopsis flammea *	CBS 324.66	C. African Rep.	AF506437	AF506437	Larsson and Larsson (2003)
* Gloiothele lamellosa *	CBS404.83	Madagascar	AF506487	AF506487	Larsson and Larsson (2003)
* Gloiothele torrendii *	JB18615	France	AF506455	AF506455	Larsson and Larsson (2003)
Lachnocladiaceae	S1PMB7	Thailand	AB365531	AB365531	Osono et al. (2009)
Lachnocladiaceae	S335WS151	Thailand	AB365532	AB365532	Osono et al. (2009)
Lachnocladium cf. brasiliense	CALD 161213-1	Espírito Santo – Brazil	**MH260037**	**MH260055**	This study
Lachnocladium cf. brasiliense	KM 57848	Puerto Rico	**MH260034**	**MH260052**	This study
			**MH260035**	**MH260053**	
			**MH260036**	**MH260054**	
* Lachnocladium schweinfurthianum *	KM 49740	Cameroon	**MH260033**	**MH260051**	This study
*Lachnocladium sp.*	KHL10556	Jamaica	AF506461	AF506461	Larsson and Larsson (2003)
*Lachnocladium sp.*	BK171002-23	Belize	DQ154110	DQ154110	Unpublished
* Metulodontia nivea *	NH13108	Russia	AF506423	AF506423	Larsson and Larsson (2003)
* Parapterulicium subarbusculum *	FLOR 56456	Rio de Janeiro – Brazil	**MH260026**	**MH260048**	This study
* Parapterulicium subarbusculum *	FLOR 56459	Rio de Janeiro – Brazil	**MH260027**	**MH260049**	This study
			**MH260028**		
			**MH260029**		
			**MH260030**		
			**MH260031**		
* Peniophora incarnata *	NH10271	Denmark	AF506425	AF506425	Larsson and Larsson (2003)
* Peniophora nuda *	FPL4756	–	–	AF287880	Hibbett et al. (2000)
* Scytinostroma alutum *	CBS 762.81	France	–	AF393075	Binder and Hibbett (2002)
* Scytinostroma caudisporum *	CBS 746.86	Gabon	–	AY293210	Binder et al. (2005)
* Scytinostroma portentosum *	EL11-99	Sweden	AF506470	AF506470	Larsson and Larsson (2003)
* Vararia insolita *	CBS 667.81	Ivory Coast	–	AF518665	Hibbett and Binder (2002)
* Vararia investiens *	TAA161422	Norway	AF506484	AF506484	Larsson and Larsson (2003)
* Vesiculomyces citrinus *	EL53-97	Sweden	AF506486	AF506486	Larsson and Larsson (2003)

## Results

### Phylogenetic analysis

**Figure 1.**
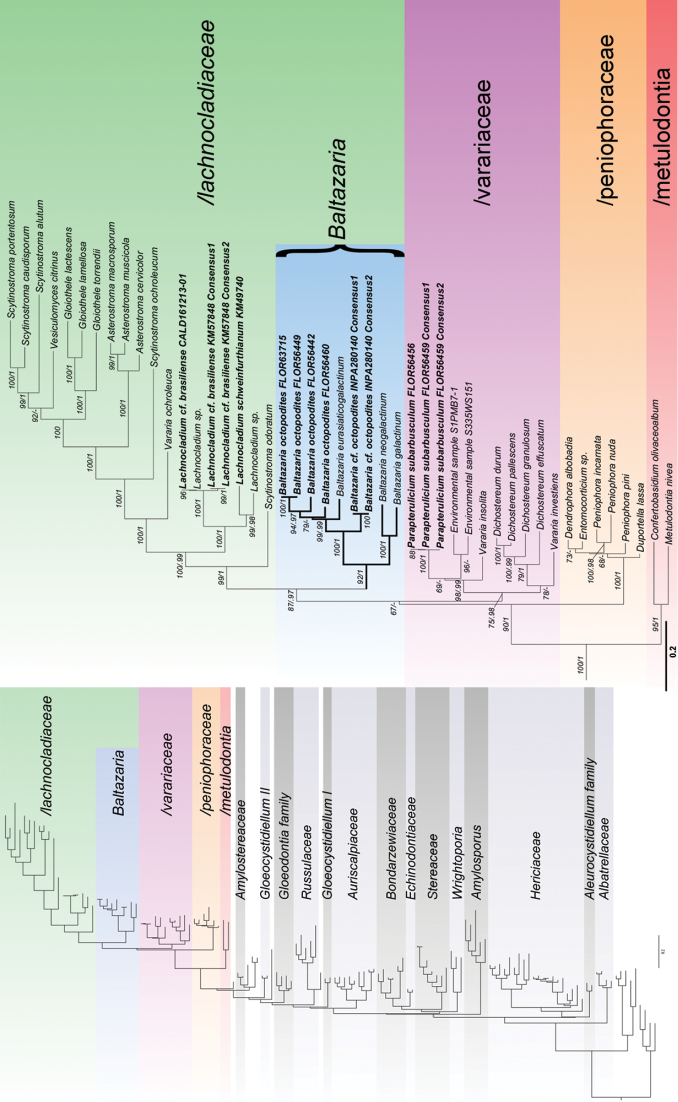
Maximum likelihood tree of Russulales on the left with /peniophorales amplified on the right. Support values on the branches are BS (>65) / BPP (>0.95), names in bold represent the newly generated sequences for this study and bold lines show the new genus. Details for the complete tree can be found in Suppl. material 1: SuppFigs 2, 3.

A total of 37 sequences were generated in this study (19 nrITS and 18 nrLSU). The final alignment consisted of 135 sequences with 2295 characters. The BI analysis converged all runs as indicated by the effective sample sizes (ESS) of all parameters above 2000 and the potential scale reduction factors (PSRF) equal 1.000 for all the parameters. The two *Parapterulicium* species were placed with strong support into /peniophorales *sensu*Larsson and Larsson (2003) as shown in the Russulales tree (Fig. 1).

The clade /peniophorales recovered in the Russulales tree and the genera which it comprises are consistent with the neighbour-joining analyses of Larsson and Larsson (2003). However, the ML tree presented here shows better resolution of the sub-clades.

Five main clades highlighted in Fig. 1 are /lachnocladiaceae (previously /asterostromataceae), *Baltazaria*, /varariaceae, /peniophoraceae and /metulodontia.

*Clade /lachnocladiaceae* (*BS=99; BPP=1*)

*Lachnocladium* formed a well-supported clade with *Scytinostroma*, *Vesiculomyces* E. Hagstr., *Gloiothele* Bres., *Asterostroma* Massee, *Varariaocholeuca* (Bourdot & Galzin) Donk, *Scytinostromaochroleucum* Donk, *Scytinostromaodoratum* (Fr.) Donk and *Baltazaria* gen. nov. (BS=99; BPP=1).

*Baltazaria* (*BS=92; BPP=1*)

This clade represents the newly proposed genus (see below). It contains the sequences of *P.octopodites*, *S.eurasiaticogalactinum*, *S.neogalactinum* and *S.galactinum*. The presence of *P.octopodites* here rendered *Parapterulicium* paraphyletic necessitating reclassification.

*Clade /varariaceae* (*BS=75; BPP=0.98*)

This clade includes *Parapteruliciumsubarbusculum*, *Dichostereum* Pilát and *Vararia* P. Karst. The inclusion of *Parapterulicium* sequences enhanced support for this clade, which was also recovered by Binder et al. (2005). The monophyly of *Dichostereum* typified by *D.durum* (Bourdot & Galzin) Pilát is strongly supported (BS=98; BPP=1). However, *Vararia* was rendered paraphyletic and will require a more thorough investigation to resolve its classification.

*Parapteruliciumsubarbusculum*, the type species of the genus, was nested within a strongly supported clade, which also contains *Varariainsolita* Boidin & Lanq. (BS=98; BPP=0.99). The reclassification of *Varariainsolita* may be warranted if future data support its placement here. *Parapteruliciumsubarbusculum* is also clustered with environmental sequences derived from subtropical leaf litter in Thailand (Osono et al. 2009). *Parapterulicium* spp. are not known outside of South America, but this suggests this species may be more widespread in subtropical and tropical regions than presently acknowledged.

*Clade /peniophoraceae.* (*BS=100; BPP=1*)

The clade /peniophoraceae includes *Peniophora*, *Duportella* Pat., *Dendrophora* (Parmasto) Chamuris and *Entomocorticium* H.S. Whitney, Bandoni & Oberw. These genera require special attention for detailed morphological and molecular studies to resolve the paraphyly of *Peniophora*, by either proposing new genera or synonymising *Dendrophora* and *Entomocorticium*. In all analyses performed in this study, there was no clear resolution for this group.

*Clade /metulodontia* (*BS=95; BPP=1*)

The clade contains *Metulodontia* Parmasto and *Confertobasidium* Jülich. Following Larsson and Larsson (2003), this well supported clade was recovered in all analyses performed.

### Taxonomy

#### Parapterulicium subarbusculum

Taxon classificationFungiAgaricalesPterulaceae

Corner, Ann. Bot., 16: 288 (1952)

Fig. 2

##### Description.

Basidiomes coralloid/filiform, up to 35 mm high, branched, erect, monoaxial with adventitious branches, yellow (10YR 8/6), solitary or gregarious. Stipe up to 13 × 0.3–0.7 mm, glabrous, concolorous with the rest of the basidiomes, attached to a small resupinate base up to 3 mm wide. Branches up to 1.3 × 0.2 mm, tapering upwards, rarely with branchlets.

Habitat: On dead twigs, petioles, leaves or seeds in the forest.

Hyphal system dimitic. Generative hyphae up to 7 μm wide thin-walled, without clamps. Skeletal hyphae 2–7 μm wide, thick-walled (up to 1.3 μm), rarely branched. Abundant dextrinoid dichophyses, up to 30 μm wide, slightly thick-walled (0.5–1 μm), branching with filiform ends, tips less than 0.5 μm wide.

Resupinate patch not well-developed in the studied material but with abundant dichophyses.

Basidia not observed.

Gloeocystidia up to 65 μm long, clavate to lanceolate/subulate, thin-walled, with numerous internal droplets, IKI-.

Basidiospores (12–)13.4–16.8(–17) × 3–3.5 μm (n = 19), hyaline, smooth, elongate, subfusiform, apex obtuse, base acute with small apiculus (0.3 μm), thin-walled and slightly amyloid, scarce in all the collected samples.

##### Specimens examined.

Brazil. Rio de Janeiro: Rio de Janeiro, Parque Nacional da Tijuca, close to Casa do Pesquisador, growing on the ground in rainforest litter, 24-25 Nov 2014, C.A. Leal-Dutra 108, 109, 117,118, 119, 120, 121, 122 (topotypes designated here: RB 639457, RB 639458, RB 639462, RB 639463, FLOR 56456, FLOR 56457, FLOR 56458, FLOR 0056459).

##### Distribution.

Brazil. Rio de Janeiro: Rio de Janeiro (Corner 1952a, Type)

##### Notes.

**Figure 2.**
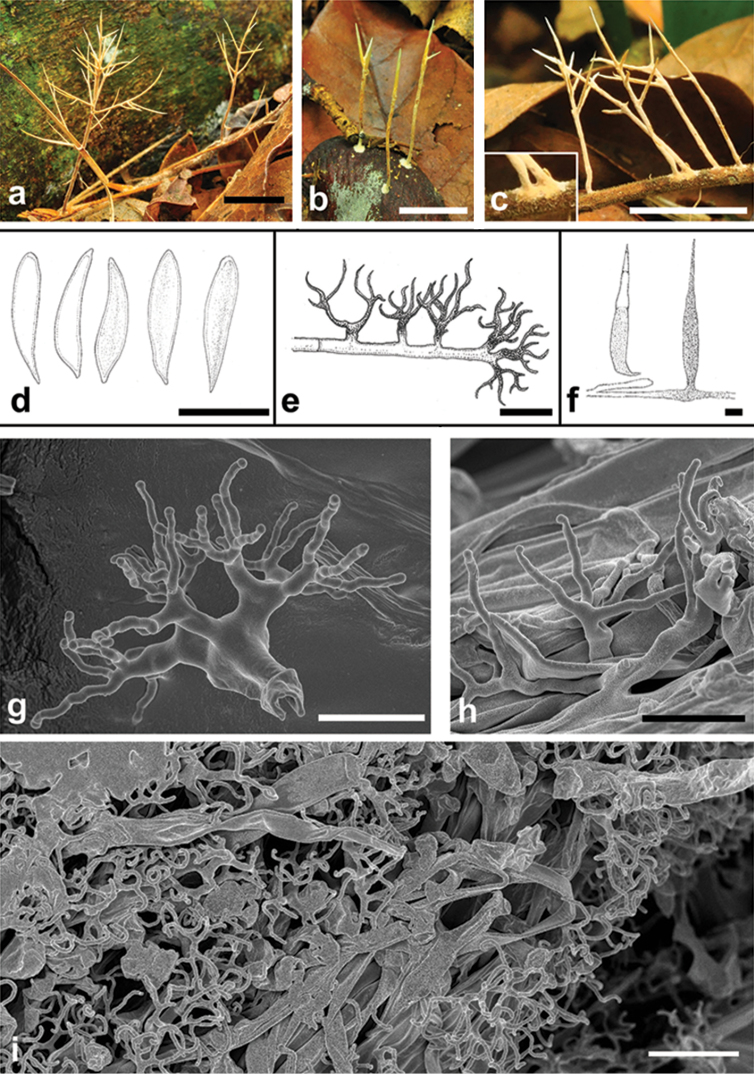
*Parapteruliciumsubarbusculum*: **a–c** basidiomes in the field. The detail in **c** shows the developing corticioid patch **d** basidiospores **e** dichophyses **f** gloeocystidia **g, h**SEM images of dichophyses; i. SEM images of basidiome surface with abundant dichophyses. Scale bars: **a–c** = 1 cm; **d–f, i** = 10 μm; **g, h** = 5 μm.

This species is recognised in the field by its characteristic resupinate disc at the base of the stipe (Fig. 2b, c). Corner (1952a) described *P.subarbusculum* from a single specimen collected in November 1948 on Corcovado in Rio de Janeiro and, based on its coralloid habit and dimitic hyphal system, placed the genus in Pterulaceae. The presence of gloeocystidia, slightly amyloid spores and dextrinoid dichophyses corroborates its placement in Russulales. It appears to be relatively common, though apparently overlooked.

#### Baltazaria

Taxon classificationFungiAgaricalesPterulaceae

C.A. Leal-Dutra, Dentinger & G.W. Griff. gen. nov.

MB825233

##### Etymology.

In honour of Dr. Juliano Marcon Baltazar, Brazilian mycologist and authority on neotropical corticioid fungi.

##### Type species.

*Baltazariagalactina* (Fr.) C.A. Leal-Dutra, Dentinger & G.W. Griff.

##### Diagnosis.

Basidiomes corticioid, adherent to effused, coriaceous/membranaceous when fresh, hard when dry, usually white, cream or pale ochraceous. Context densely homogeneous with thick-walled and dextrinoid skeletal-binding hyphae, sometimes bearing rows of short papillae or skeletodendrohyphidia. Global distribution.

##### Notes.

**Figure 3.**
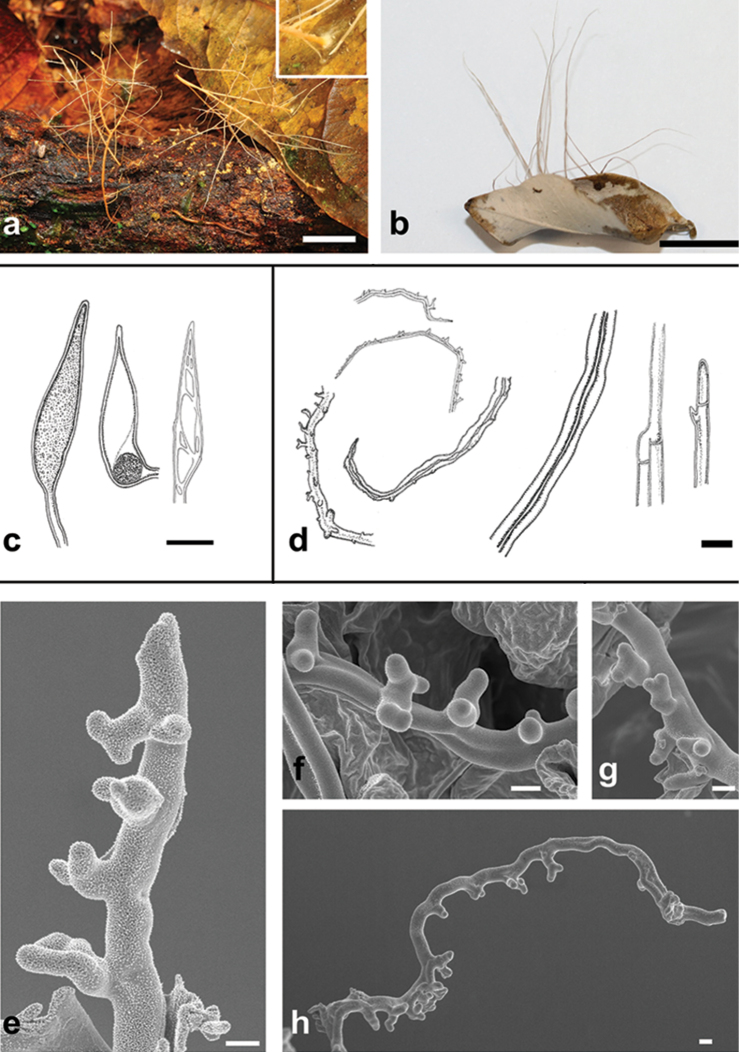
*Baltazariaoctopodites*: **a, b** basidiomes in the field (INPA280140 and FLOR56460), the detail in **a** shows the anchorage point in the leaf, the whitish resupinate area in **b** shows the corticioid portion of the fungus **c** gloeocystidia **d** skeletal hyphae, skeletal hyphae inflated in KOH (third from the right) and generative hyphae (first and second from the right) **e–h**SEM images of papillate skeletal hyphae. Scale bars: **a–b** = 1 cm; **c–d** = 10 μm; **e–h** = 1 μm).

The diagnosis of Boidin and Lanquetin (1987) for *Scytinostromaeurasiaticogalactinum* and *S.neogalactinum* describes both species with the same morphological characters as *S.galactinum* (Fr.) Donk but with reproductive incompatibility between the species and different distributions. In the discussion on the *S.galactinum* complex, the authors mention the branched skeletal hyphae that starts with conspicuous 2–3 branched short projections and then become longer, a feature resembling the *Parapteruliciumoctopodites* papillate skeletal hyphae (Fig. 3d–h). Moreover, the description of *S.galactinum* by Lentz and Burdsall (1973) mentions the hymenium with conspicuous skeletodendrohyphidia. However, Bernicchia and Gorjón (2010) claimed the species does not present dendrohyphae; instead, the authors describe the presence of skeletal-binding hyphae. It is likely that the papillate skeletal hyphae described by Corner (1952a), the short and branched projections described by Boidin and Lanquetin (1987) and the skeletodendrohyphidia described by Lentz and Burdsall (1973), are nothing more than early developmental stages of the skeletal-binding hyphae described by Bernicchia and Gorjón (2010).

#### Baltazaria galactina

Taxon classificationFungiAgaricalesPterulaceae

(Fr.) C.A. Leal-Dutra, Dentinger & G.W. Griff. comb. nov.

MB825235

##### Basionym.

*Thelephoragalactina* Fr., Nova Acta R. Soc. Scient. upsal., Ser. 3 1(1): 136 (1851) [1855]. ≡ *Corticiumgalactinum* (Fr.) Moffatt, Bulletin of the Nat. Hist. Surv. Chicago Acad. Sci. 7(1): 137 (1909). ≡ *Scytinostromagalactinum* (Fr.) Donk, Fungus, Wageningen 26: 20 (1956).

= *Thelephorasuaveolens* Moug. ex Fr., Elench. fung. (Greifswald) 1: 208 (1828).

= *Stereumsuaveolens* (Moug. ex Fr.) Fr., Epicr. syst. mycol. (Upsaliae): 553 (1838) [1836-1838].

= *Xerocarpussuaveolens* (Moug. ex Fr.) P. Karst., Bidr. Känn. Finl. Nat. Folk 37: 137 (1882).

Description in Lentz and Burdsall (1973).

#### Baltazaria eurasiaticogalactina

Taxon classificationFungiAgaricalesPterulaceae

(Boidin & Lanq.) C.A. Leal-Dutra, Dentinger & G.W. Griff. comb. nov.

MB825236

##### Basionym.

*Scytinostromaeurasiaticogalactinum* Boidin & Lanq., Biblthca Mycol. 114: 57 (1987)

Description in Boidin and Lanquetin (1987).

#### Baltazaria neogalactina

Taxon classificationFungiAgaricalesPterulaceae

(Boidin & Lanq.) C.A. Leal-Dutra, Dentinger & G.W. Griff. comb. nov.

MB825237

##### Basionym.

*Scytinostromaneogalactinum* Boidin & Lanq., Biblthca Mycol. 114: 59 (1987).

Description in Boidin and Lanquetin (1987).

#### Baltazaria octopodites

Taxon classificationFungiAgaricalesPterulaceae

(Corner) C.A. Leal-Dutra, Dentinger & G.W. Griff. comb. nov.

MB825234

Fig. 3

##### Basionym.

*Parapteruliciumoctopodites* Corner, Ann. Bot., 16: 286 (1952)

##### Description.

Basidiomes resupinate (Fig. 3b), 0.1–0.5 mm thick, membranaceous, covering leaves and twigs, hymenophore smooth, white (2.5Y 8/2) to pale yellow (2.5Y 8/4), forming rhizomorph-like structures up to 7 cm high and scarcely to profusely branched that may be confused with coralloid basidiomes (Fig. 3a, b).

Substrate: On dead twigs and leaves.

Hyphal system dimitic, profusely interwoven. Generative hyphae 2–5 μm wide, thin-walled, without clamps. Skeletal hyphae 2–6 μm (up to 10 μm in KOH) wide, walls dextrinoid, up to 1.5 μm thick, strongly swelling in KOH (up to 4.5 μm). Termini of hymenial skeletal hyphae papillate, presenting short protuberances 2–10 × 1.5–2.5 μm, sometimes ramified resembling skeletodendrohyphidia.

Putative hymenium with abundant basidioles up to 25 × 6 μm, clavate, growing immersed in the papillate hyphae.

Gloeocystidia up to 80 × 8–14 μm, clavate to lanceolate, thin-walled, densely multiguttulate or with abundant granular contents. Present in all parts of the basidiomes, including the corticioid form.

Basidiospores and basidia not observed.

##### Specimens examined.

Brazil. Rio Grande do Sul: no date, J. Rick (holotype: BPI 333063). São Paulo: Apiaí, Parque Estadual Turístico do Alto Ribeira, growing on the ground in rainforest litter, 14-15 Dec. 2014, M.A. Reck 1003/14, 1069/14 (FLOR 56442, FLOR 56449). Santa Catarina: Florianópolis, UCAD, 9 Jan. 2015, G. Flores 14 (FLOR 56460). Paraná: Foz do Iguaçú, Parque Nacional do Iguaçú, Trilha da torre, 22 Jan. 2017, C.A.T. Oliveira 160 (FLOR 63715). Amazonas: Rio Preto da Eva, ARIE-PDBFF - Reserva do Km 41, 17 Mar. 2017, C.A. Leal-Dutra, L.A. Clasen, Q.V. Montoya, O. Pereira 170309-26 (INPA 280140).

##### Distribution.

Brazil. Rio Grande do Sul: São Leopoldo (Corner, 1952a; Type). São Paulo: Apiaí. Santa Catarina: Florianópolis. Paraná: Foz do Iguaçu. Amazonas: Rio Preto da Eva (this study).

##### Notes.

The dimitic hyphal system, the papillate surface at the ends of the skeletal hyphae and the gloeocystidia agree perfectly with Corner’s original descriptions (Corner 1952a). Corner (1952a) described this species from a collection where no fertile structures were observed; the new collections were also sterile. As no spores or fertile basidia were found, the term putative hymenium is given to the region with abundant basidiole-like structures. Furthermore, the lack of sexual characters observed in *B.octopodites*, combined with the undeveloped binding-skeletal hyphae, might indicate that this species is only known by young basidiomes or non-reproductive growth forms (i.e. explorative rhizomorphs). This is the first record of *B.octopodites* from the States of Amazonas, Paraná, Santa Catarina and São Paulo.

## Discussion

The /lachnocladiaceae clade was named /asterostromataceae by Larsson and Larsson (2003), who also proposed a deeper molecular study involving *Lachnocladium* to find the exact placement of the genus. In this study, *Lachnocladium* spp., typified by *L.brasiliense* (Lév.) Pat., formed a strongly supported (BS=99; BPP=1) clade with the previously called /asterostromataceae, which includes *Scytinostroma*, *Vesiculomyces*, *Gloiothele*, *Asterostroma*, *Varariaocholeuca*, *Scytinostromaochroleucum*, *Scytinostromaodoratum* and the new genus *Baltazaria*. Thus, we decided to name the clade /lachnocladiaceae to suggest the need for a thorough study on the morphology of these genera to re-circumscribe Lachnocladiaceae. Binder et al. (2005) recovered this clade but did not include *Lachnocladium*. *Scytinostroma*, typified by *S.portentosum* (Berk. & M.A. Curtis) Donk, forms a clade with robust support with *S.caudisporum* Boidin, Lanq. & Gilles and *S.alutum* Lanq. (BS=99; BPP=1), meaning the other species of *Scytinostroma* sampled in this study (*S.ochroleucum*, *S.odoratum*, *S.eurasiaticogalactinum*) require reclassification. Monophyly of *Asterostroma* and *Gloiothele* is also strongly supported (BS=100; BPP=1), including the type species *A.apalum* (Berk. & Broome) Massee (= *A.muscicola*) and *Gloiothelelamellosa* (Henn.) Bres., respectively.

Future studies of Lachnocladiaceae may recommend *Baltazaria* be classified in its own family. However, we view our study as incomplete and it would therefore be premature to erect a new family at this time.

The most distinctive feature of *B.octopodites* is the papillate skeletal hyphae that form one or two rows of short, round and sometimes branched projections, similar to some skeletodendrohyphidia of *B.galactinum* (Lentz and Burdsall 1973). Another notable characteristic of this species is the hyphal swelling seen in KOH, which is also found in some species of *Peniophora* Cooke, *Dichostereum* and *Vararia* (Stalpers 1996; Stalpers and Buchanan 1991). In addition, the multigutullate gloeocystidia present in *P.subarbusculum* and *B.octopodites* might have the same origin as those in *Russula* Pers. and *Auriscalpium* Gray, which were shown by McLaughlin et al. (2008) to be a likely synapomorphy of Russulales. Taken together, alongside the molecular evidence presented in this study, these corroborating morphological features add strong support to the reclassification of these fungi and suggest that aforementioned hyphal features may be unifying characters for /peniophorales.

All collections of *B.octopodites* made to date are sterile with no spores or basidia observed. Although the hymenium might have been missed due to developmental idiosyncrasies, such as ephemeral nocturnal production (Corner 1950, McLaughlin and McLaughlin 1972), the function of the filiform projections, believed to be coralloid basidiomes, may not be for sexual reproduction. Instead, they may function as exploratory appendages, similar to mycelial cords and rhizomorphs in other fungi (e.g. *Crinipellis* Pat./*Marasmius* Fr., *Armillaria* (Fr.) Staude etc.) or as a strategy for binding substrate materials together (Cairney 1991; Hedger et al. 1993; Snaddon et al. 2011). This characteristic, combined with the fact that no spores have been reported, raises the possibility that an independent sexual form, similar to the resupinate basidiomes of *Scytinostroma*, may exist. Considering these assumptions, *B.octopodites* might be more common than previously thought, since it is probably overlooked during fieldwork, mistakenly identified as a rhizomorph.

A third species of *Parapterulicium*, *P.simplex*, is still known only from type material originally collected in Argentina (Corner 1957). It would be prudent to include this species in a full revision of the genus, which would require targeted fieldwork at the type locality. We anticipate that, despite the rarity of their documentation, these filiform fungi are abundant and widespread.

## Supplementary Material

XML Treatment for Parapterulicium subarbusculum

XML Treatment for Baltazaria

XML Treatment for Baltazaria galactina

XML Treatment for Baltazaria eurasiaticogalactina

XML Treatment for Baltazaria neogalactina

XML Treatment for Baltazaria octopodites

## Supplementary materials

Supplementary material 1 Species used in the Russulales analyses and their GenBank accession numbers of nrITS and nrLSU sequences Data type: species data This is an open access article distributed under the terms of the Creative Commons Attribution License (CC BY 4.0), which permits unrestricted use, distribution, and reproduction in any medium, provided the original author and source are credited.Caio A. Leal-Dutra, Maria Alice Neves, Gareth W. Griffith, Mateus A. Reck, Lina A. Clasen, Bryn T. M. Dentinger
