# Supplementary material for: Reclassification of Parapterulicium Corner (Pterulaceae, Agaricales), contributions to Lachnocladiaceae and Peniophoraceae (Russulales) and introduction of Baltazaria gen. nov
Source: MycoKeys. 2018 Jul 31;(37):39–56. doi: 10.3897/mycokeys.37.26303 (PMC6081468; doi:10.3897/mycokeys.37.26303)
Supplement: Supplementary material 1 — Species used in the Russulales analyses and their GenBank accession numbers of nrITS and nrLSU sequences [file mycokeys-37-039-s001.docx]

**Supplementary file 1**

| **SuppTable 1.** Species used in the Russulales analyses and their GenBank accession numbers of nrITS and nrLSU sequences. Newly generated sequences are shown in bold. | | | | |
| --- | --- | --- | --- | --- |
| **Species** | **Sample no.** | **Locality** | **GenBank Accession no.** | |
|  |  |  | **ITS** | **LSU** |
| *Albatrellus ovinus** | PV 22-89 | Czech Republic | AF506396 | AF506396 |
| *Albatrellus subrubescens** | PV 154-95 | Czech Republic | AF506395 | AF506395 |
| *Aleurocystidiellum disciforme** | NH 13003 | Russia | AF506402 | AF506402 |
| *Aleurocystidiellum subcruentatum** | NH 12874 | Germany | AF506403 | AF506403 |
| *Aleurodiscus amorphus** | KHL 4240 | Sweden | AF506397 | AF506397 |
| *Amylonotus africanus** | Ipulet F1883 | Uganda | KJ807070 | KJ807083 |
| *Amylonotus labyrinthinus** | Yuan 1475 | China | KM107860 | KM107878 |
| *Amylosporus bracei** | 1008/77 | USA | KM267724 | KJ807076 |
| *Amylosporus campbellii** | Gilbertson 14806 | USA | KM107861 | KM107879 |
| *Amylosporus casuarinicola** | Dai 6914 | China | KJ807068 | – |
| *Amylosporus rubellus** | Dai 9233 | China | KJ807071 | KJ807084 |
| *Amylosporus succulentus** | Dai 7802 | China | KM213669 | KM213671 |
| *Amylostereum areolatum** | NH 8041 | Romania | AF506405 | AF506405 |
| *Amylostereum laevigatum** | NH 2863 | Sweden | AF506407 | AF506407 |
| *Asterostroma cervicolor* | KHL9239 | Puerto Rico | AF506408 | AF506408 |
| *Asterostroma macrosporum* | TMI 25697 | Japan | NR119394 | – |
| *Asterostroma muscicola* | TMI 25860 | Japan | AB439551 | AB439551 |
| *Auricularia mesenterica** | EL 66-97 | USA | AF506492 | AF506492 |
| *Auriscalpium vulgare** | EL 33-95 | Sweden | AF506375 | AF506375 |
| *Baltazaria eurasiaticogalactina* | CBS 666.84 | France | – | AY293211 |
| *Baltazaria galactina* | NH4863 | Sweden | AF506466 | AF506466 |
| *Baltazaria neogalactina* | *CBS 758.86* | France | – | – |
| *Baltazaria octopodites* | FLOR 56442 | São Paulo - Brazil | **MH260024** | **MH260043 MH260044 MH260045 MH260046** |
| *Baltazaria octopodites* | FLOR 56449 | São Paulo - Brazil | **MH260025** | **MH260047** |
| *Baltazaria octopodites* | FLOR 56460 | Santa Catarina - Brazil | **MH260032** | **MH260050** |
| *Baltazaria octopodites* | FLOR 63715 | Paraná - Brazil | **MH260042** | **MH260060** |
| *Baltazaria octopodites* | INPA 280140 | Amazonas - Brazil | **MH260038 MH260039 MH260040 MH260041** | **MH260056 MH260057 MH260058 MH260059** |
| *Basidioradulum radula** | NH 9453 | Finland | AF347105 | AF347105 |
| *Boidinia aculeata** | Wu 890714-52 | China | AF506433 | AF506433 |
| *Boidinia granulata** | Wu 9209-34 | China | AY048880 | AY048880 |
| *Boidinia propinqua** | KHL 10931 | Jamaica | AF506379 | AF506379 |
| *Bondarzewia montana** | DAOM 415 | Canada | DQ200923 | DQ234539 |
| *Bondarzewia podocarpi** | Dai 9261 | China | KJ583207 | KJ583221 |
| *Byssoporia terrestris** | Hjm 18172 | Sweden | DQ389664 | DQ389664 |
| *Confertobasidium olivaceoalbum* | FP90196 | USA | AF511648 | AF511648 |
| *Dendrophora albobadia* | TDeAB1029 | – | AF119522 | AF119522 |
| *Dentipellicula leptodon** | GB 11123 | Uganda | EU118625 | EU118625 |
| *Dentipellicula taiwaniana** | Cui 8346 | China | JQ349114 | JQ349100 |
| *Dentipellis coniferarum** | Cui 10063 | China | JQ349106 | JQ349092 |
| *Dentipellis fragilis** | Dai 9009 | China | JQ349108 | JQ349094 |
| *Dentipellis microspora** | Cui 10035 | China | JQ349112 | JQ349098 |
| *Dentipellis parmastoi** | Cui 8513 | China | JQ349113 | JQ349099 |
| *Dentipellopsis dacrydicola** | Dai 12004 | China | JQ349104 | JQ349089 |
| *Dentipratulum bialoviesense** | GG 1645 | France | AF506389 | AF506389 |
| *Dichostereum durum* | FG1985 | France | AF506429 | AF506429 |
| *Dichostereum effuscatum* | GG930915 | France | AF506390 | AF506390 |
| *Dichostereum granulosum* | NH7137/696 | Canada | AF506391 | AF506391 |
| *Dichostereum pallescens* | NH7046/673 | Canada | AF506392 | AF506392 |
| *Duportella lassa* | SP6129 | Russia | KJ509191 | KJ509191 |
| *Echinodontium ryvardenii** | Ryvarden 43370 | Italy | AF506431 | AF506431 |
| *Echinodontium sulcata** | KHL 8267 | Russia | AF506414 | AF506414 |
| *Echinodontium tinctorium** | NH 6695 | Canada | AF506430 | AF506430 |
| *Entomocorticium sp.* | FL_19 | USA | KJ620518 | KJ620518 |
| *Exidia glandulosa** | EL Mar-97 | Sweden | AF506493 | AF506493 |
| *Exidia recisa** | EL 15-98 | Sweden | AF347112 | AF347112 |
| *Gloeocystidiellum bisporum** | KHL 11135 | Norway | AY048877 | AY048877 |
| *Gloeocystidiellum clavuligerum** | NH 11185 | Spain | AF310088 | AF310088 |
| *Gloeocystidiellum compactum** | Wu 880615-21 | China | AF506434 | AF506434 |
| *Gloeocystidiellum formosanum** | Wu 9404-16 | China | AF506439 | AF506439 |
| *Gloeocystidiellum porosum** | NH 10434 | Denmark | AF310094 | AF310094 |
| *Gloeocystidiopsis cryptacanthus** | KHL 10334 | Puerto Rico | AF506442 | AF506442 |
| *Gloeocystidiopsis flammea* | CBS 324.66 | C. African Rep. | AF506437 | AF506437 |
| *Gloeodontia columbiensis** | NH 11118 | Spain | AF506444 | AF506444 |
| *Gloeodontia discolor** | KHL 10099 | Puerto Rico | AF506445 | AF506445 |
| *Gloeodontia pyramidata** | Ryvarden 15502 | Colombia | AF506446 | AF506446 |
| *Gloeodontia subasperispora** | KHL 8695 | Norway | AF506404 | AF506404 |
| *Gloeopeniophorella convolvens** | KHL 10103 | Puerto Rico | AF506435 | AF506435 |
| *Gloiodon nigrescens** | Desjardin 7287 | Bali | AF506450 | AF506450 |
| *Gloiodon strigosus** | JS 26147 | Norway | AF506449 | AF506449 |
| *Gloiothele lactescens** | EL8-98 | Sweden | AF506453 | AF506453 |
| *Gloiothele lamellosa* | CBS404.83 | Madagascar | AF506487 | AF506487 |
| *Gloiothele torrendii* | JB18615 | France | AF506455 | AF506455 |
| *Hericium abietis** | NH 6990 | Canada | AF506456 | AF506456 |
| *Hericium alpestre** | NH 13240 | Russia | AF506457 | AF506457 |
| *Hericium americanum** | DAOMF-21467 | Canada | AF506458 | AF506458 |
| *Hericium cirrhatum** | Tübingen F794 | Germany | AF506385 | AF506385 |
| *Hericium coralloides** | NH 282 | Sweden | AF506459 | AF506459 |
| *Hericium erinaceus** | NH 12163 | Russia | AF506460 | AF506460 |
| *Heterobasidion annosum** | Korhonen 06129/6 | Russia | KJ583211 | KJ583225 |
| *Heterobasidion parviporum** | Korhonen 04121/3 | Finland | KJ583212 | KJ583226 |
| Lachnocladiaceae | S1PMB7 | Thailand | AB365531 | AB365531 |
| Lachnocladiaceae | S335WS151 | Thailand | AB365532 | AB365532 |
| *Lachnocladium cf. brasiliense* | CALD 161213-1 | Espírito Santo - Brazil | **MH260037** | **MH260055** |
| *Lachnocladium cf. brasiliense* | KM 57848 | Puerto Rico | **MH260034 MH260035MH260036** | **MH260052 MH260053 MH260054** |
| *Lachnocladium schweinfurthianum* | KM 49740 | Cameroon | **MH260033** | **MH260051** |
| *Lachnocladium sp.* | KHL10556 | Jamaica | AF506461 | AF506461 |
| *Lachnocladium sp.* | BK171002-23 | – | DQ154110 | DQ154110 |
| *Lactarius leonis** | SJ 91016 | Sweden | AF506411 | AF506411 |
| *Larssoniporia incrustatocystidiata** | Dai 13607 | China | KM107863 | KM107880 |
| *Larssoniporia tropicalis** | Ryvarden 45363 | Belize | KJ513294 | KJ807089 |
| *Laxitextum bicolor** | NH 5166 | Sweden | AF310102 | AF310102 |
| *Lentinellus auricula** | KGN 280994 | Sweden | AF506415 | AF506415 |
| *Lentinellus cochleatus** | KGN 96-09-28 | Sweden | AF506417 | AF506417 |
| *Lentinellus omphalodes** | JJ 2077 | Sweden | AF506418 | AF506418 |
| *Lentinellus ursinus** | EL 73-97 | USA | AF506419 | AF506419 |
| *Lentinellus vulpinus** | KGN 98-08-25 | Sweden | AF347097 | AF347097 |
| *Megalocystidium luridum** | KHL 8635 | Norway | AF506422 | AF506422 |
| *Metulodontia nivea* | NH13108 | Russia | AF506423 | AF506423 |
| *Parapterulicium subarbusculum* | FLOR 56456 | Rio de Janeiro - Brazil | **MH260026** | **MH260048** |
| *Parapterulicium subarbusculum* | FLOR 56459 | Rio de Janeiro - Brazil | **MH260027 MH260028 MH260029 MH260030 MH260031** | **MH260049** |
| *Peniophora incarnata* | NH10271 | Denmark | AF506425 | AF506425 |
| *Peniophora nuda* | FPL4756 | – | – | AF287880 |
| *Peniophora pini** | Hjm 18143 | Sweden | EU118651 | EU118651 |
| *Polyporoletus sublividus** | JA 30918 | – | DQ389663 | DQ389663 |
| *Pseudowrightoporia crassihypha** | Yuan 6247 | China | KM107873 | KM107892 |
| *Pseudowrightoporia cylindrospora** | Ryvarden 46609 | USA | KJ513290 | KJ807079 |
| *Pseudowrightoporia hamata** | Dai 8152 | China | KM107869 | KM107888 |
| *Pseudowrightoporia japonica** | KUC 20110908 | Korea | KC166692 | KC166692 |
| *Pseudowrightoporia oblongispora** | Yuan 6106 | China | KM107867 | KM107886 |
| *Pseudoxenasma verrucisporum** | EL 34-95 | Sweden | AF506426 | AF506426 |
| *Russula violacea** | SJ 93009 | Sweden | AF506465 | AF506465 |
| *Scytinostroma alutum* | CBS 762.81 | France | – | AF393075 |
| *Scytinostroma caudisporum* | CBS 746.86 | Gabon | – | AY293210 |
| *Scytinostroma ochroleucum** | TAA 159869 | Australia | AF506468 | AF506468 |
| *Scytinostroma odoratum** | KHL 8546 | Sweden | AF506469 | AF506469 |
| *Scytinostroma portentosum* | EL11-99 | Sweden | AF506470 | AF506470 |
| *Scytinostromella nannfeldtii** | NH 7476 | Norway | AF506472 | AF506472 |
| *Sistotrema brinkmannii** | NH 11412 | Turkey | AF506473 | AF506473 |
| *Sistotrema coronilla** | NH 7598 | Canada | AF506475 | AF506475 |
| *Sistotrema muscicola** | KHL 8791 | Sweden | AF506474 | AF506474 |
| *Sistotrema sernanderi** | KHL 8576 | Sweden | AF506476 | AF506476 |
| *Stereum hirsutum** | NH 7960 | Romania | AF506479 | AF506479 |
| *Trichaptum abietinum** | NH 12842 | Finland | AF347104 | AF347104 |
| *Vararia insolita* | CBS 667.81 | Ivory Coast | – | AF518665 |
| *Vararia investiens* | TAA161422 | Norway | AF506484 | AF506484 |
| *Vararia ochroleuca** | JS 24400 | Norway | AF506485 | AF506485 |
| *Vesiculomyces citrinus* | EL53-97 | Sweden | AF506486 | AF506486 |
| *Wrightoporia avellanea** | LR 41710 | Jamaica | AF506488 | AF506488 |
| *Wrightoporia lenta** | Dai 12850 | China | KM107874 | KM107893 |
| *Wrightoporia subavellanea** | Dai 11484 | China | KJ513295 | KJ807085 |
| *Wrightoporiopsis amylohypha** | Yuan 3460 | China | KM107875 | KM107894 |
| *Wrightoporiopsis biennis** | Cui 8457 | China | KJ807066 | KJ807074 |
| *Sequences used from Chen et al. (2016). | | | | |

**Agaricomycetidae dataset analysis**

For a broad phylogenetic placement, a multiple sequence alignment containing the nrLSU sequences of 886 Agaricomycetidae taxa was created by adding the sequences generated in this study to the dataset of Moncalvo et al. (2002) as adapted by (Dentinger and McLaughlin 2006). The dataset was aligned using the L-INS-i algorithm in MAFFT v7.311 (Katoh and Standley 2013). The alignments were examined and adjusted manually using MEGA 7 (Kumar et al. 2016).

Maximum likelihood (ML) analysis was performed using RAXML v8 (Stamatakis 2014) under a GTRGAMMA. The Best-score ML tree was calculated from 100 independent ML searches using the option *-f d*. The statistical support of clades in the best-tree were assessed non-parametric bootstrap (option *-f b*) with 10000 replicates. One sequence of *Auricularia nigricans* (Sw.) Birkebak, Looney & Sánchez-García was used as outgroup.


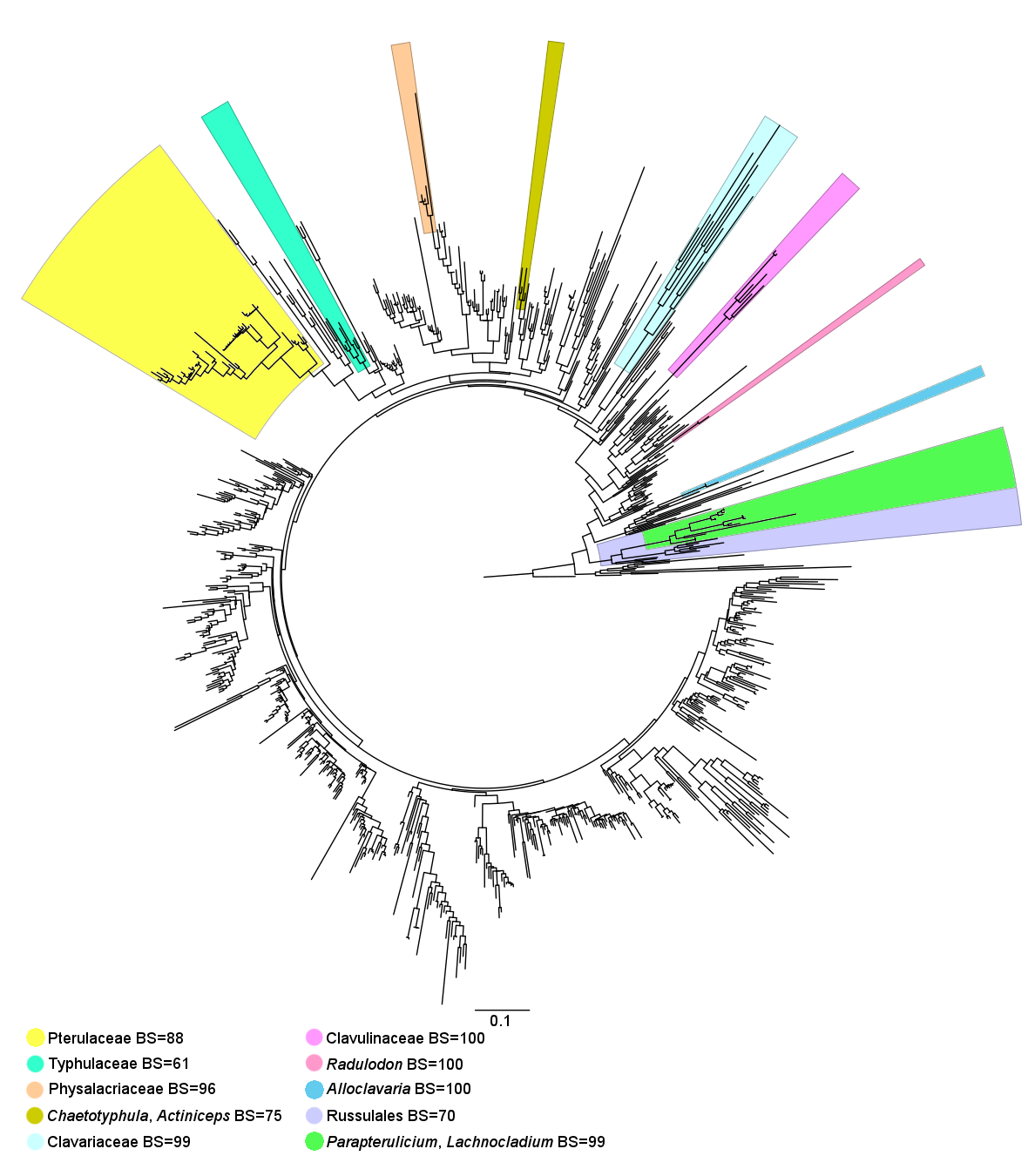


**SuppFigure 1**: Agaricomycetidae Best-score ML tree showing the position of Parapterulicium (green) into Russulales (lilac). Other highlighted clades indicates the position of other coralloid/clavarioid taxa.


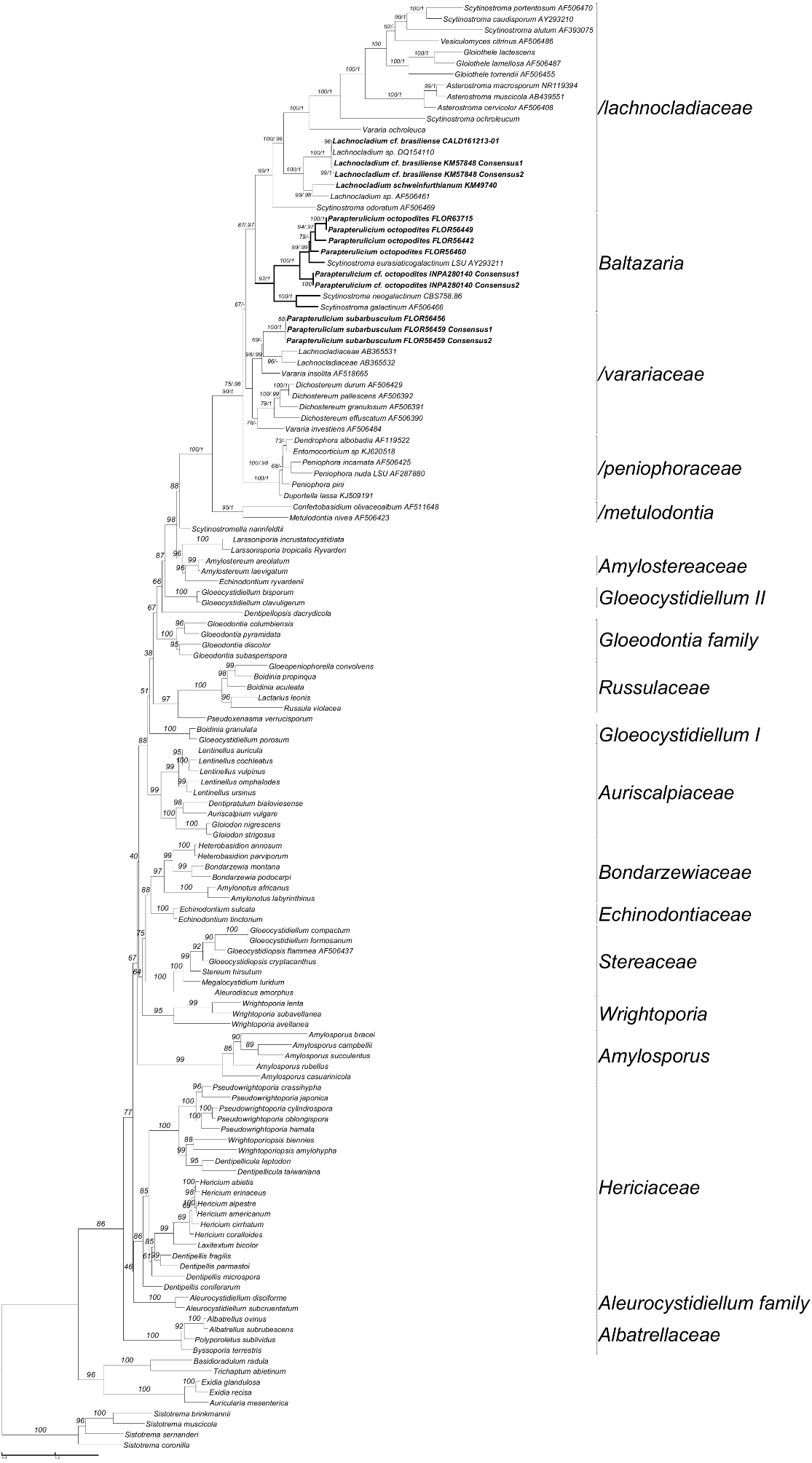


**SuppFigure 2:** Full best-score ML tree of Russulales with bootstrap support (1000 replicates).


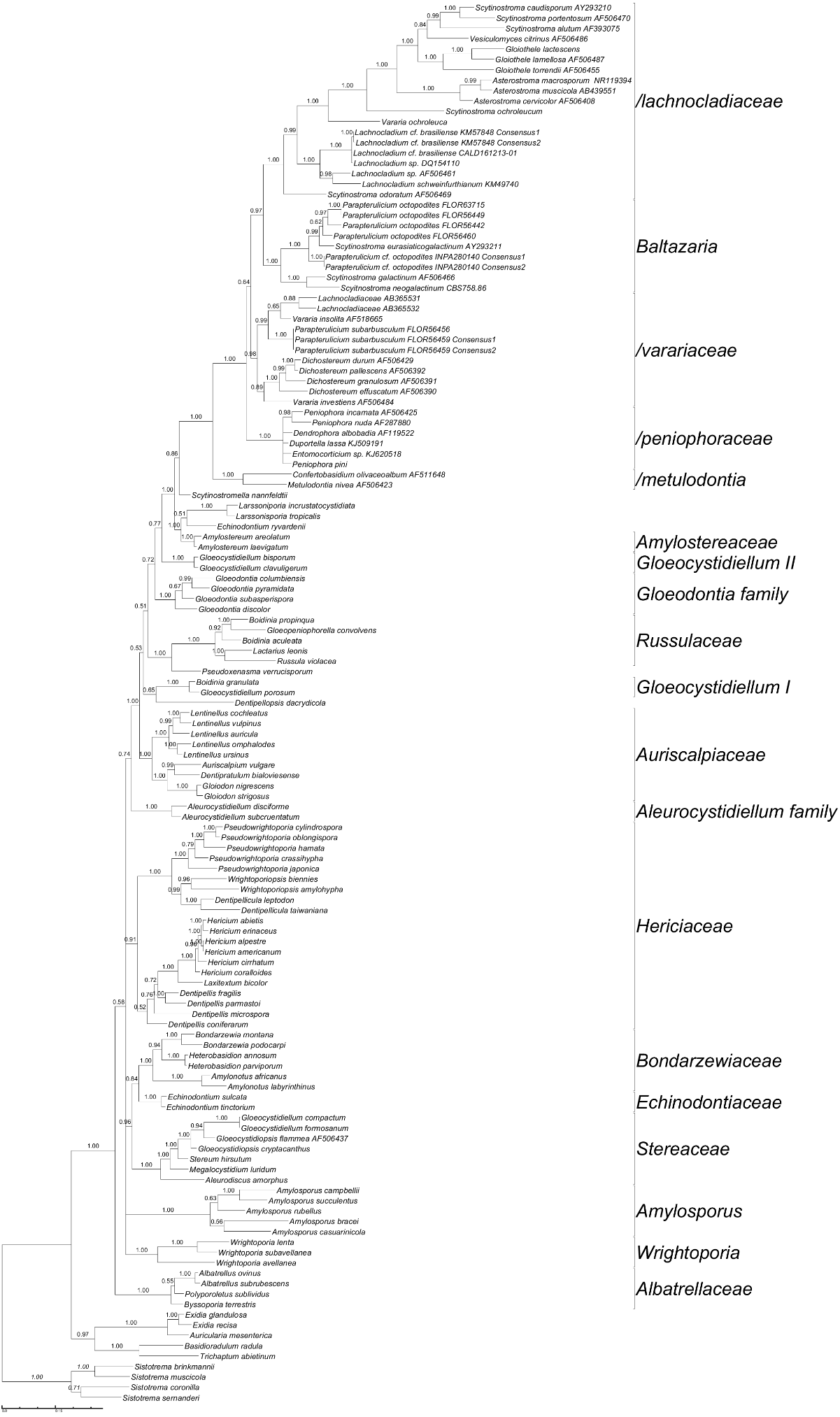


**SuppFigure 3:** Bayesian Inference tree topology with Bayesian Posterior Probability support values.

**References**

Dentinger BTM, McLaughlin DJ (2006) Reconstructing the Clavariaceae using nuclear large subunit rDNA sequences and a new genus segregated from Clavaria. Mycologia 98: 746-762.

Katoh K, Standley DM (2013) MAFFT multiple sequence alignment software version 7: improvements in performance and usability. Mol Biol Evol 30: 772-780.

Kumar S, Stecher G, Tamura K (2016) MEGA7: Molecular Evolutionary Genetics Analysis Version 7.0 for Bigger Datasets. Mol Biol Evol 33: 1870-1874.

Moncalvo J-M, Vilgalys R, Redhead SA, Johnson JE, James TY, Catherine Aime M, Hofstetter V, Verduin SJW, Larsson E, Baroni TJ, Greg Thorn R, Jacobsson S, Clémençon H, Miller OK (2002) One hundred and seventeen clades of euagarics. Molecular Phylogenetics and Evolution 23: 357-400.
